# BiOCl/TiO_2_/diatomite composites with enhanced visible-light photocatalytic activity for the degradation of rhodamine B

**DOI:** 10.3762/bjnano.10.139

**Published:** 2019-07-16

**Authors:** Minlin Ao, Kun Liu, Xuekun Tang, Zishun Li, Qian Peng, Jing Huang

**Affiliations:** 1 School of Minerals Processing and Bioengineering, Central South University, Changsha 410083, China; 2 Hunan Key Laboratory of Mineral Materials and Application, Central South University, Changsha 410083, China

**Keywords:** BiOCl, diatomite, photocatalysis, sewage treatment, TiO_2_, visible-light photocatalysis

## Abstract

A BiOCl/TiO_2_/diatomite (BTD) composite was synthesized via a modified sol–gel method and precipitation/calcination method for application as a photocatalyst and shows promise for degradation of organic pollutants in wastewater upon visible-light irradiation. In the composite, diatomite was used as a carrier to support a layer of titanium dioxide (TiO_2_) nanoparticles and bismuth oxychloride (BiOCl) nanosheets. The results show that TiO_2_ nanoparticles and BiOCl nanosheets uniformly cover the surface of diatomite and bring TiO_2_ and BiOCl into close proximity. Rhodamine B was used as the target degradation product and visible light (λ > 400 nm) was used as the light source for the evaluation of the photocatalytic properties of the prepared BTD composite. The results show that the catalytic performance of the BTD composite under visible-light irradiation is much higher than that of TiO_2_ or BiOCl alone. When the molar ratio of BiOCl to TiO_2_ is 1:1 and the calcination temperature is 400 °C, the composite was found to exhibit the best catalytic effect. Through the study of the photocatalytic mechanism, it is shown that the strong visible-light photocatalytic activity of the BTD composite results mainly from the quick migration of photoelectrons from the conduction band of TiO_2_/diatomite to the surface of BiOCl, which promotes the separation effect and reduces the recombination rate of the photoelectron–hole pair. Due to the excellent catalytic performance, the BTD composite shows great potential for wide application in the field of sewage treatment driven by solar energy.

## Introduction

In recent decades, with intense industry development and worldwide social economy growth, the discharge of wastewater has been continuously increasing. The main sources of wastewater are from the printing and textile industries, as well as from chemical fertilizers [1–2]. The wastewater from these sources is composed of organic compounds, which are usually hazardous to human beings, aquatic organisms and even nature as a whole. In recent years, advanced oxidation processes including Fenton [3], Fenton-like [4–6] and photocatalysis [7] reactions have been widely used in wastewater treatment. In addition, photocatalysis has attracted great attention due to advantages such as environmental sustainability, low-cost and ease of application, which are superior to other approaches of environmental remediation [8–9]. However, the application of photocatalysis is still hindered due to the agglomeration of photocatalyst particles, the difficulty of photocatalyst recovery and low photocatalytic performance [10].

As one of the most promising photocatalysts, in terms of its chemical stability, non-toxicity, photo-corrosion resistance in aqueous media and advanced oxidation properties, titanium dioxide (TiO_2_) has been widely studied [11–12] and employed for water splitting [13], energy storage [14], and organic pollutant degradation [15]. However, according to previous studies, one limitation to its photocatalytic activity is that the photocatalytic process mainly occurs on the surface of the photocatalyst, which is a problem because the TiO_2_ nanoparticles readily agglomerate [10]. According to the current works, this problem can be solved by fixing the TiO_2_ onto the surface of the carrier to form a uniform layer. Till now, various materials have been studied and successfully applied as the photocatalytic carrier such as kaolinite [16], diatomite [17], zeolite [18], silica nanofiber [19], etc., and have been studied and successfully applied as the photocatalytic carrier. As a result, well-dispersed, nanometer-sized TiO_2_ immobilized on diatomite is obtained in the present paper.

Even when TiO_2_ is well-dispersed, the problem of the band gap is yet another impediment to overcome. The value of the band gap of TiO_2_ is determined to be 3.20 eV [20], which means the photocatalytic process can just occur under UV-light irradiation. As we all know, it is more meaningful to make full use of the visible light spectrum in photocatalysis. To solve this problem, many effective methods have been studied such as doping [21], sensitization [22], modification [23], coupled and supported semiconductors [24]. As an important bismuth oxyhalide semiconductor material, bismuth oxychloride (BiOCl) has gained extensive attention in photocatalysis [25–26]. BiOCl has a band gap of 3.05–3.55 eV [27], which allows it to respond mainly to ultraviolet light. Over decades, the BiOCl/TiO_2_ heterostructure has been studied successfully and shows higher photocatalytic activity [28], which inspired us to load BiOCl onto the well-dispersed TiO_2_ to improve the TiO_2_/diatomite composite.

In this paper, we report a novel photocatalyst prepared by dispersing TiO_2_ and BiOCl on the surface of diatomite for the first time. Rhodamine B (RhB) is one of the most commonly used dyes and is a highly toxic compound with potential carcinogenicity. It is often used in textile, painting, chemical and other industries. RhB released from these industries is considered as an key organic pollutant due to its chemical stability, non-biodegradability, high light resistance and oxidation degradation, which may cause long-term damage to ecosystems. Therefore, the photocatalytic activity of the photocatalyst was studied under visible light with RhB as the target degradation product. In addition, the photocatalytic mechanism was further analyzed by investigating the structure and photochemical properties of the catalyst.

## Experimental

### Materials

Tetrabutyl titanate, ethanol, acetic acid, nitric acid, bismuth nitrate pentahydrate (Bi(NO_3_)_3_·5H_2_O), potassium chloride (KCl), mannitol and rhodamine B were purchased from the Chemical Reagents Co., Ltd. of the Sinopharm. All reagents were analytical grade and could be used directly without any purification. Deionized water was used throughout the experiment. In addition, diatomite was purchased from Yingkou, China, and used after removing impurities by acid leaching.

### Preparation of TiO_2_/diatomite composite

In a typical synthesis, we prepared the TiO_2_/diatomite composite via a modified sol–gel method: Firstly, we added 12 mL of tetrabutyl titanate to 20 mL ethanol under stirring, which was recorded as solution A. Solution B was obtained by mixing 4 g of diatomite, 20 mL of ethanol, 0.5 mL of glacial acetic acid and 1.5 mL of 0.1 mol/L nitric acid under stirring. In order to obtain the precursor colloid, solution B was dripped into solution A under stirring. After that, the precursor was dried at 60 °C and calcined at 600 °C for 2.5 h to prepare the TiO_2_/diatomite composite.

### Preparation of BiOCl/TiO_2_/diatomite (BTD) composite

Subsequently, 0.2 g of the TiO_2_/diatomite composite and 0.6 mmol of Bi(NO_3_)_3_·5H_2_O were dissolved in 20 mL of 0.1 mol/L mannitol simultaneously under stirring. The solution obtained by dissolving 0.12 g of KCl in 5 mL distilled water was dripped into the above mixed mannitol solution under stirring, and the resulting mixed solution was filtered to obtain a solid. After washing, drying and calcining at 400 °C for 2 h, the BiOCl/TiO_2_/diatomite (BTD) composites were prepared. For comparison purposes, pure BiOCl was prepared according to similar procedures without adding TiO_2_/diatomite.

### Characterization

The morphology of the samples was characterized by a Phenom World Phenom ProX scanning electron microscope (SEM) and a JEOL JEMe1200EX transmission electron microscopy (TEM). Both the SEM and TEM were used with an accelerating voltage of 200 kV. The X-ray diffraction (XRD) patterns of the samples were recorded by a X-ray powder diffractometer using a Cu Kα source (λ = 0.15418 nm) at a scanning rate of 2°/min between 5° and 80°. A Thermo Fisher VG Scientific VG ESCALAB250Xi electron spectrometer was used to observe X-ray photoelectron spectroscopy (XPS) results. A monochromatic Al Kα source (1486.7 eV) and a 300 × 500 μm spot size was used to collect the spectra. In 77 K nitrogen atmosphere, the specific surface area and pore size distribution of the sample were determined by a Micromeritics ASAP 2020 surface area and porosity measurement system. A Unico UV-2600 spectrophotometer was used to analyze the concentration of RhB in the photocatalytic process. The photoelectrochemical properties were analyzed using electrochemical workstations (Gamry interface 1010 and Chenhua CHI700E) with blue light (400–450 nm) irradiation. The steady-state photoluminescence (PL) spectra of the samples were detected by using a Hitachi F-7000 fluorescence spectrophotometer with excitation at 280 nm.

### Photocatalytic activity tests

With visible-light irradiation, the photocatalytic activity was tested by the degradation of RhB. 100 mL of RhB (10 mg/L) solution was used as a degradation object and 0.05 g of BTD was added to the RhB solution. In order to achieve adsorption–desorption equilibrium, the solution was stirred for 60 min in a dark environment. Subsequently, we illuminated the RhB solution with a 400 W Xe lamp with a cut-off filter (λ ≥ 400 nm for visible light irradiation). The residual RhB concentration was determined with a UV–vis spectrophotometer using a previously published method [29] over a given time interval. In addition, the adsorption capacity and photocatalytic activity of TiO_2_/diatomite and pure BiOCl were analyzed by the same method.

In addition, cyclic experiments were carried out to prove the recyclability of photocatalysts. The photocatalyst was collected by centrifugation and the surface organic matter was removed by ethanol and water washing several times. After these treatments, the photocatalyst was applied to the degradation of fresh RhB.

## Results and Discussion

### Structural analysis

**Figure 1:**
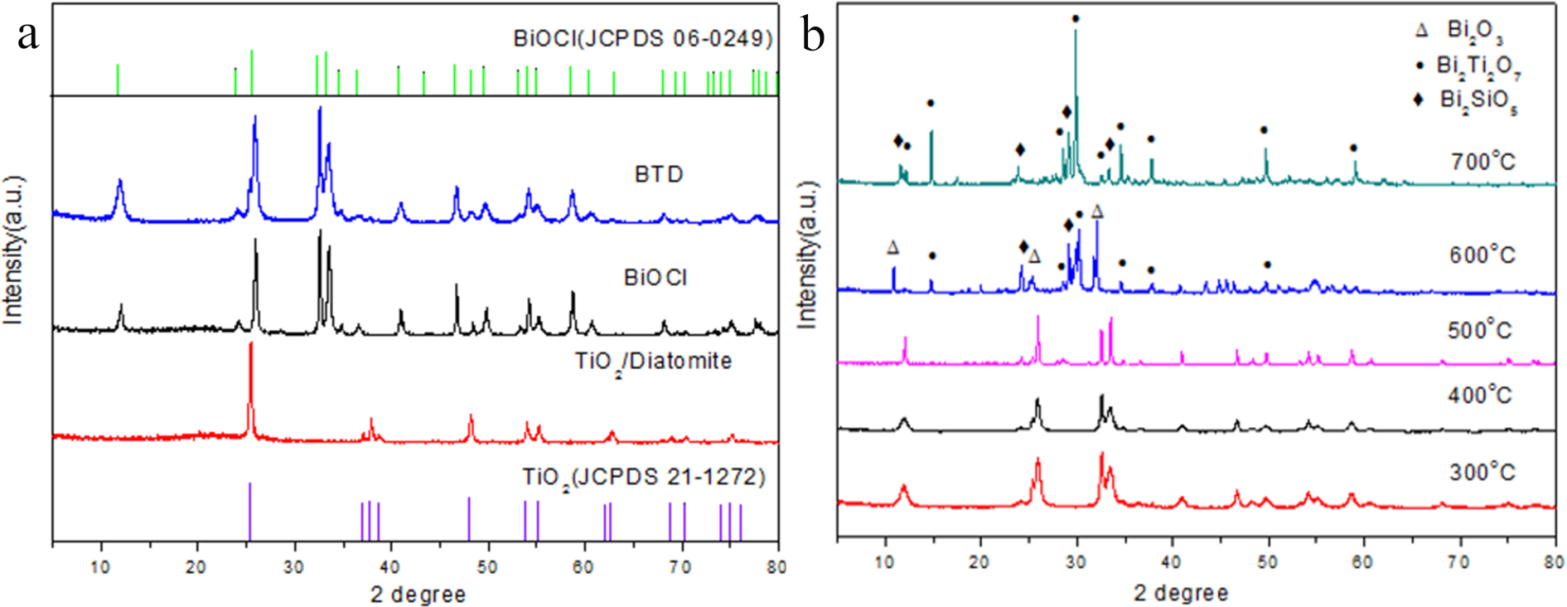
XRD patterns of the TiO_2_/diatomite, BiOCl and BTD (a) and BTD with different calcination temperatures (b).

XRD was used to analyze the phase structure and crystal structure of the samples. The results are shown in Figure 1a. The diffraction peaks of the prepared BiOCl are in good agreement with the standard XRD data of JCPDS No.06-0249. In the pattern of TiO_2_/diatomite, an inconspicuous broad peak ranging from 15 to 25° shows the amorphous nature of diatomite, and other obvious peaks at 25.3°, 37.8°, 48°, 53.9° and 55.1° coincide well with anatase TiO_2_ (JCPDS No.21-1272). The average crystal size of TiO_2_ is 25.5 nm, which is calculated by the Scherrer equation [30]. The XRD pattern of BTD shows sharp BiOCl and TiO_2_ diffraction peaks, indicating a high degree of crystallization of BTD. In addition, when the calcination temperature is higher than 500 °C, BiOCl will gradually change to Bi_2_O_3_, and Bi_2_SiO_5_ and Bi_2_Ti_2_O_7_ will be formed (Figure 1b). At the same time, TiO_2_ will gradually change from anatase to rutile, resulting in significant degradation of photocatalytic properties [31]. We speculate that the existence of BiOCl leads to the change of the crystal transition temperature of TiO_2_ in the composites. This speculation is based on previous reports that some modification methods may lead to crystal transformation and grain size change [27,32].

**Figure 2:**
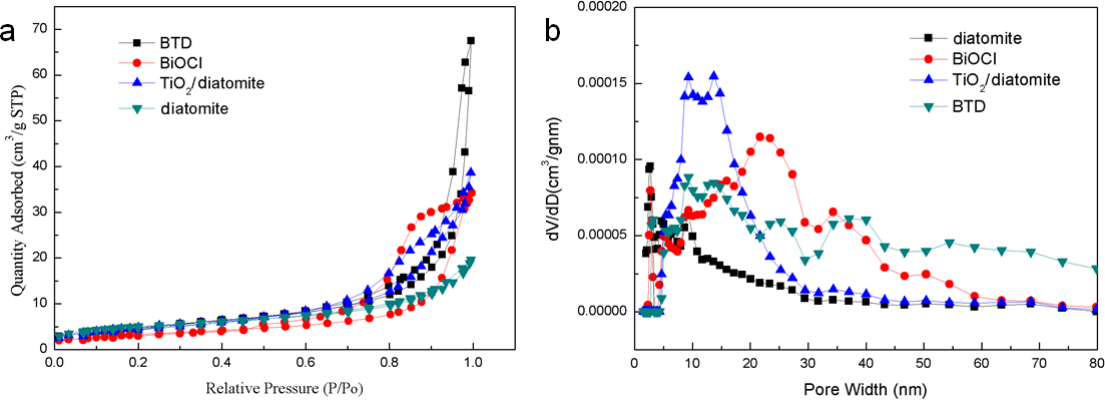
N_2_ adsorption–desorption isotherms (a) and pore size distribution plots (b) of the samples.

The photocatalytic activity of the catalyst is related to its specific surface area. Generally, the larger the specific surface area, the higher the photocatalytic activity [33]. The specific surface area, pore size and pore volume of the samples were determined by N_2_ adsorption–desorption measurements. The results of BiOCl, diatomite, TiO_2_/diatomite and BTD are shown in Figure 2. In addition, the data of specific surface area and pore volume are shown in Table 1. Density functional theory (DFT) mode was undertaken to characterize the porosity of these samples. According to the N_2_ adsorption–desorption isotherms, we can see that all four samples belong to IV-type isotherms. BiOCl has an H2-type hysteresis loop, while diatomite, TiO_2_/diatomite and BTD have an H4-type hysteresis loop, indicating that all samples have a mesoporous structure. At the same time, the pore size distribution and average pore size also confirm that the samples have mesoporous structure. Figure 2b shows that the pore size distribution of BTD has a wide size distribution, although most are mesoporous, and the rest are macroporous. The specific surface area of BTD is slightly lower than that of diatomite due to the surface loading of BiOCl and TiO_2_. The pore volume of the BTD composite is larger than that of BiOCl and TiO_2_/diatomite, which indicates that BiOCl and TiO_2_ are mainly dispersed on the surface of diatomite, but not in the pores. The larger pore volume will be beneficial to the enrichment and degradation of dyes, thus showing a higher catalytic performance [33].

**Table 1:** Specific surface area and pore volume of BiOCl, diatomite, TiO_2_/diatomite and BTD.

Sample	BET specific surface area (m^2^/g)	Pore volume (cm^3^/g)	Avgerage diameter (nm)

diatomite	20.99	0.03	6.95
BiOCl	12.19	0.05	17.37
TiO_2_/diatomite	17.73	0.06	13.49
BTD	17.80	0.10	23.44

**Figure 3:**
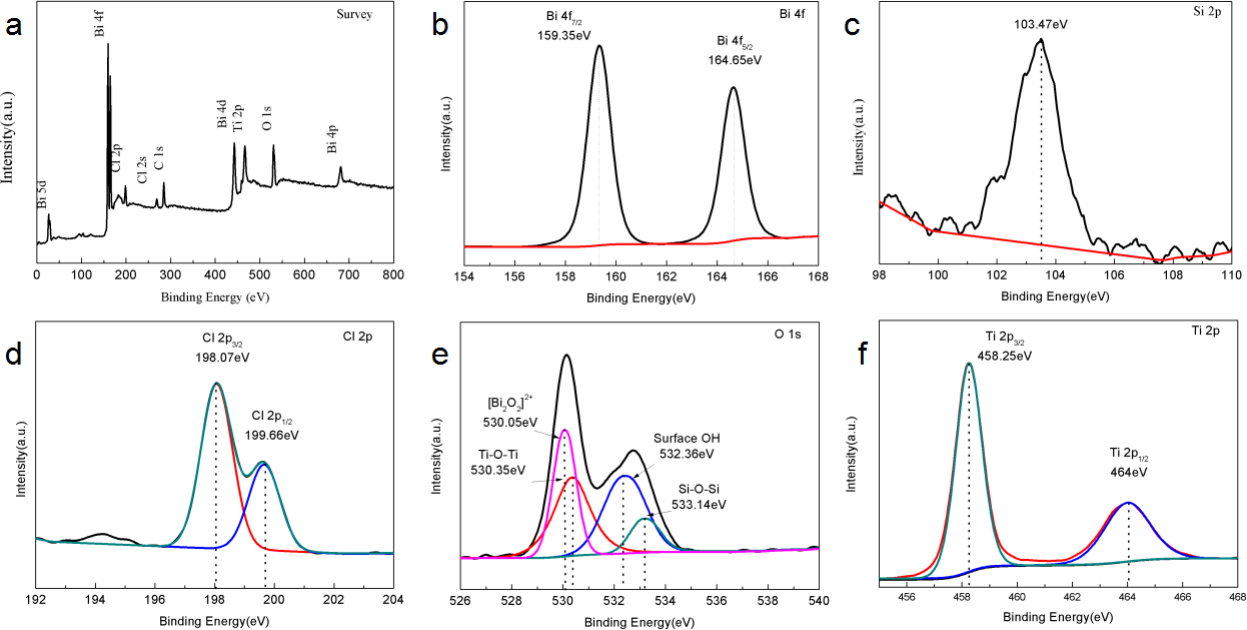
XPS spectra of BTD, including the survey scan (a) and detailed Bi 4f (b), Si 2p (c), Cl 2p (d), O 1s (e) and Ti 2p (f) spectra.

The surface chemical state of BTD was characterized by XPS. The survey spectrum (Figure 3a) clearly shows the signals of Ti, O, Cl, Bi and Si. The two peaks corresponding to 159.35 and 164.65 eV belong to Bi 4f_7/2_ and Bi 4f_5/2_ respectively in the Bi 4f spectrum [34] (Figure 3b), indicating that Bi^3+^ is the main chemical state of Bi in the composites. In the Si 2p spectrum (Figure 3c), the main peak at 103.47 eV is attributed to diatomite [29]. The Cl 2p spectrum (Figure 3d) shows peaks with a binding energy of 198.07 and 199.66 eV, which can be respectively ascribed to Cl 2p_3/2_ and Cl 2p_1/2_ [35]. The deconvolution of the O 1s spectrum (Figure 3e) shows four peaks located at 530.05, 530.35, 532.36 and 533.14 eV, corresponding to [Bi_2_O_2_]^2−^, Ti–O–Ti, surface OH and Si–O–Si [29], respectively. This confirms that BiOCl, TiO_2_ and SiO_2_ exist on the surface of BTD. In addition, the two peaks at 458.25 and 464 eV in the Ti 2p spectrum (Figure 3f) belong to Ti 2p_3/2_ and Ti 2p_1/2_, respectively [36]. In addition, no other chemical contact points have been found for the time being, which is similar to other reports on BiOCl/TiO_2_ [29,37]. The XPS results of recovered BTD (Figure S1, Supporting Information File 1) show that the signals of Ti, O, Cl, Bi and Si can still be found in the survey spectrum. In addition, in the spectrum of Bi 4f, Si 2p, Cl 2p, O 1s, Ti 2p, the position of the peak has minor deviation, but the peak shape has not changed, which indicates that the BTD has not changed after photocatalysis.

### Morphology analysis

**Figure 4:**
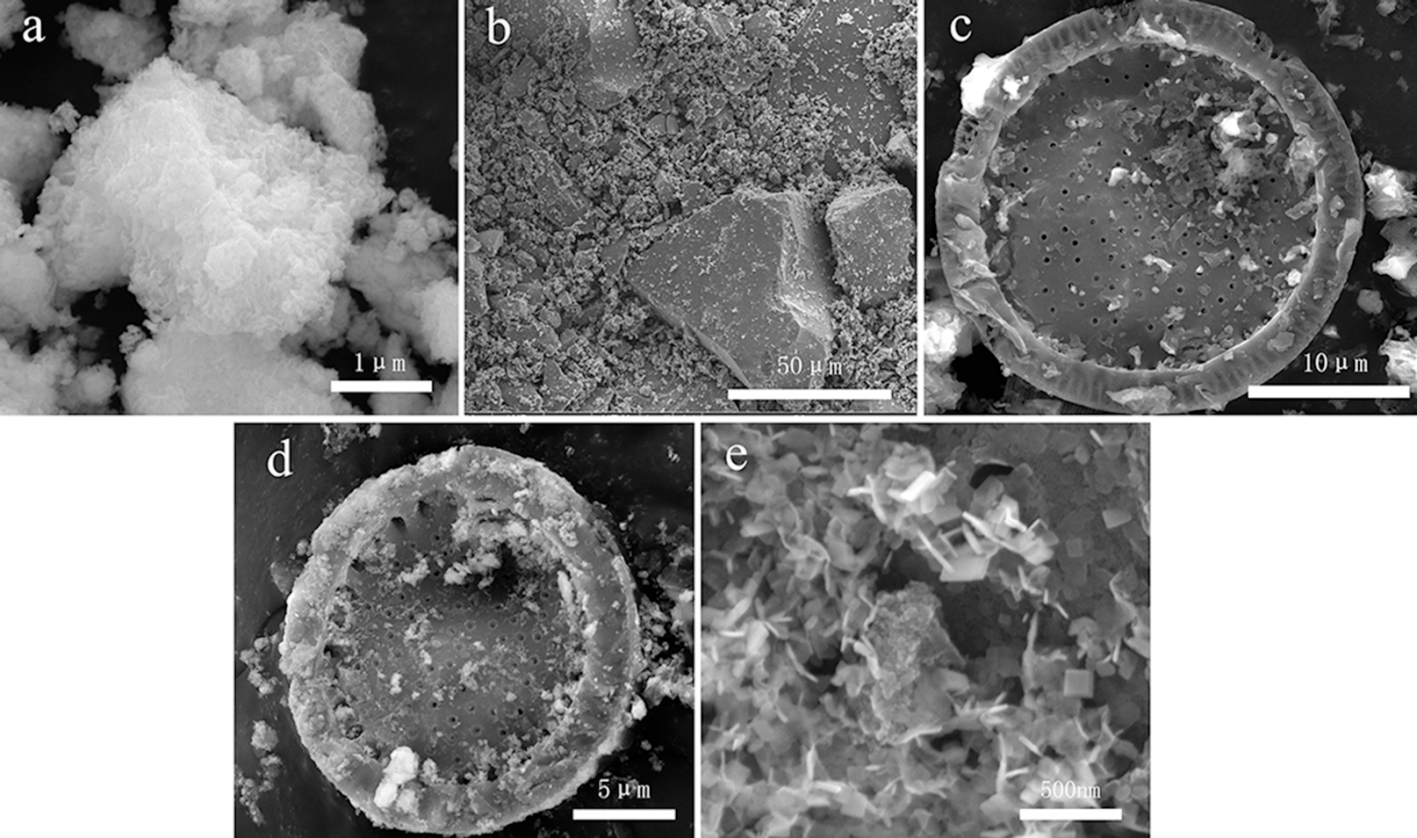
SEM images of BiOCl (a), TiO_2_ (b), TiO_2_/diatomite (c) and BTD (d and e).

**Figure 5:**
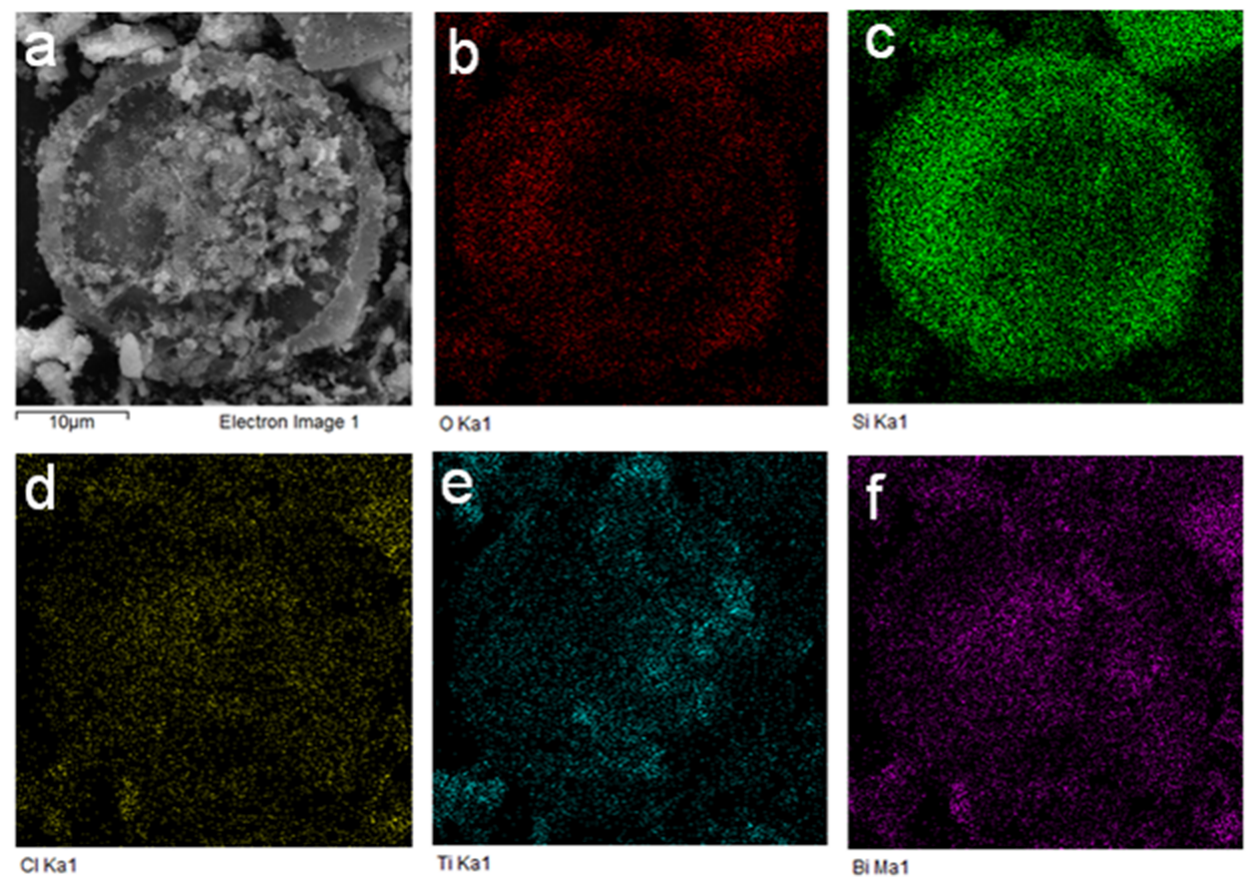
Energy-dispersive X-ray spectroscopy (EDS) mapping of BTD, including SEM image (a), O element (b), Si element (c), Cl element (d), Ti element (e) and Bi element (f).

Scanning electron microscopy (SEM) was used to study the morphology of the samples. The BiOCl sample is composed of lamellar crystals with poor dispersity, presenting an agglomeration morphology (Figure 4a). The SEM results of pure TiO_2_ show that the poor dispersion of the TiO_2_ nanoparticles leads to agglomeration, as shown in Figure 4b. Figure 4c shows that diatomite in TiO_2_/diatomite maintains a disc morphology with many tiny TiO_2_ particles distributed on it without agglomeration. In the BTD composite (Figure 4d and 4e), the disc-like morphology of diatomite is still intact, and abundant and well-dispersed TiO_2_ nanoparticles and BiOCl nanosheets can be found on the surface. Elemental mapping was carried out by energy-dispersive X-ray spectroscopy (EDS) to study the elemental dispersion on the BTD surface. Figure 5 shows that the elements Bi, Si, Cl, O and Ti distribute uniformly on the diatomite disc, which confirms the successful synthesis of the BTD composite.

**Figure 6:**
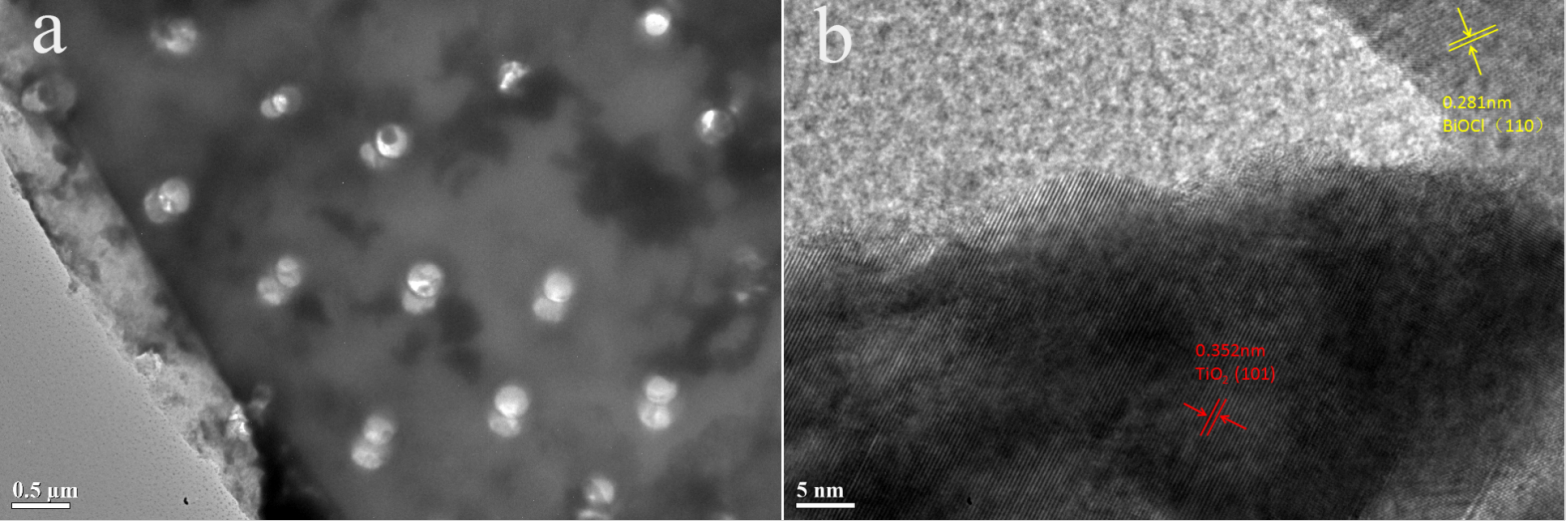
TEM image (a) and high-resolution image showing the lattice fringes (b) of BTD.

In order to further observe the morphology, TEM images of BTD were taken. Figure 6a clearly shows the disc-like porous morphology of diatomite in BTD, while a large number of granular and flaky substances (TiO_2_ and BiOCl) can be seen on the surface, which is in good agreement with the SEM results. The obvious lattice fringes can be observed in Figure 6b, indicating that the crystallinity of the composites is improved. The lattice fringes of 0.352 nm and 0.281 nm are matched with anatase TiO_2_ (101) and BiOCl (110) planes, respectively. The results show that diatomite in BTD is tightly connected with TiO_2_ and BiOCl, which mainly acts as a carrier to solve the problem of agglomeration of TiO_2_ and BiOCl.

### Photocatalytic activity analysis

**Figure 7:**
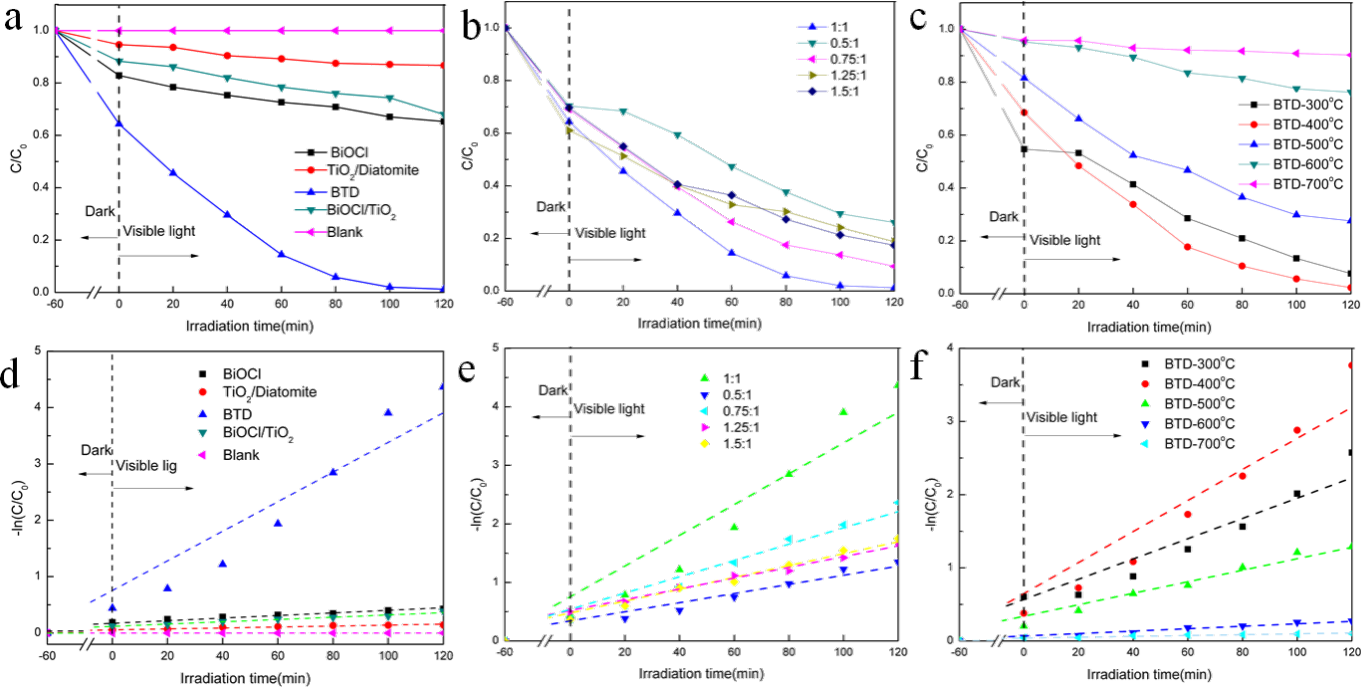
The photodegradation curves and corresponding kinetic plots of different samples, including various photocatalysts (a and d), BTD with different molar ratios of BiOCl to TiO_2_ (b and e), and BTD prepared with different calcination temperatures (c and f).

The photocatalytic degradation of RhB under visible-light irradiation was studied in this work. As shown in Figure 7a, RhB does not self-degrade under visible light and the BTD composite obviously has higher photocatalytic activity than its individual constitutes alone or in other combinations. After two hours of illumination, BTD showed good photocatalytic activity, and the residual concentration of RhB was close to zero. The results show that BTD has obvious advantages over pure BiOCl and other complexes. As shown in Figure 7b, the sample with a BiOCl to TiO_2_ molar ratio of 1:1 shows the best visible-light photocatalytic activity, and at the same time, the highest crystallinity and the most active sites were found. Figure 7c shows the comparison of the photocatalytic activity of BTD prepared from different calcination temperatures, clearly indicating that the BTD calcined at 400 °C is the best. When the calcined temperature exceeds 400 °C, the photocatalytic activity of the obtained BTD decreases continuously, which is mainly results from the transformation of TiO_2_ and BiOCl at higher temperatures (e.g., TiO_2_ transforms from anatase to rutile, and BiOCl also transforms to Bi_2_O_3_ with the generation of Bi_2_SiO_5_ and Bi_2_Ti_2_O_7_). The corresponding kinetic curves are shown in Figure 7d,e,f. The degradation of RhB conforms to the pseudo-first-order kinetics model [38]: ln *C*/*C*_0_ = *kt*, where the apparent reaction rate constant and degradation time are expressed by *k* and *t*, and the initial concentration of RhB and the concentration at transit time *t* are represented by *C*_0_ and *C*, respectively.

Supporting Information File 1, Figure S2a shows the UV–vis spectra of RhB in the degradation process by BTD. It can be seen that with the increase of illumination time, the absorbance intensity decreases and the peak shifts toward shorter wavelength (blue shift). This is mainly due to de-ethylation of RhB, accompanied by the breakage of aromatic groups. This is a process of constant transformation into small molecules [39]. Total organic carbon (TOC), representing the total amount of organic matter in water by the amount of carbon, is a comprehensive indicator for rapid verification [40]. Supporting Information File 1, Figure S2b shows the change of TOC/TOC_0_ with the increase of reaction time. The results show that the organic matter content in water decreases with the increase of illumination time, which is in accordance with the results mentioned above. Compared with the results of UV–vis and TOC, it can be seen that RhB has not been completely mineralized, although it has been completely decolorized under the same conditions. The result shows that about 45% of carbon is degraded to CO_2_.

Photoelectrons and holes in photocatalysts have strong reductive and oxidative abilities, so they react with oxygen and other substances to form a variety of active species [41]. The generation of particular active species in the photocatalytic process varies with the type of catalyst (mainly the energy band structure) [42]. KI, IPA and 1,4-benzoquinone (BQ) were used as scavengers for pores (h^+^), hydroxyl radicals (OH) and superoxide radicals (O_2_^−^), respectively, to determine the active substances in the photocatalytic process. As a reference, the degradation of RhB was performed under the same conditions without any scavengers. The results (Supporting Information File 1, Figure S3a) show that the presence of BQ inhibits the degradation of RhB, indicating that ·O_2_^−^ is the main active substance in the photocatalytic process. Secondly, the presence of KI and IPA also inhibits the degradation of RhB, but the inhibition effect is slightly less. It has been proved that the h^+^ and ·OH are also active species but are not as active as O_2_^−^ in the photodegradation process. The recyclability of the photocatalysts is of great significance for the evaluation of photocatalytic performance and practical application [43]. Five cycles of photocatalytic activity of the BTD composite were investigated and the results (Supporting Information File 1, Figure S3b) show that BTD has good cyclic ability and stability.

### Photocatalytic mechanism analysis

**Figure 8:**
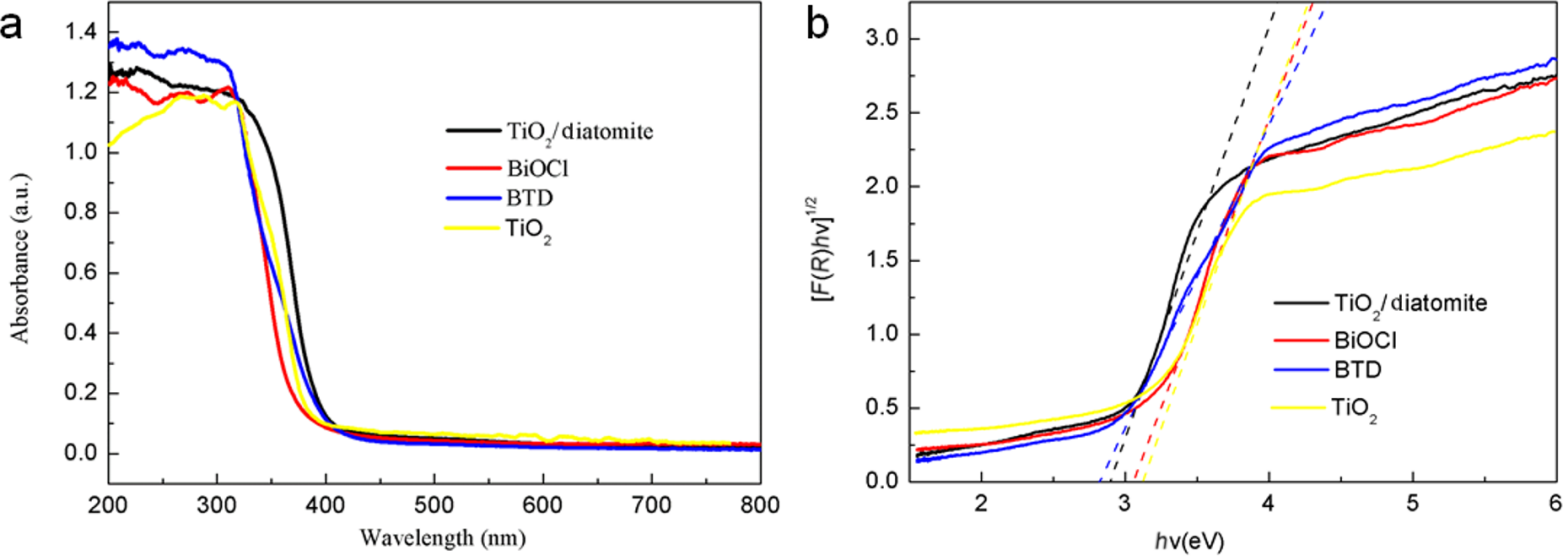
UV–vis absorption spectra (a) and band gap energy (b) of various samples.

In order to reveal the photocatalytic mechanism, we observe the optical, photochemical and electrochemical properties to study the energy band structure and carrier migration pathway of BTD. Figure 8a presents the UV–vis diffuse reflectance spectra of TiO_2_, BiOCl, TiO_2_/diatomite, and BTD. The results show that TiO_2_, BiOCl, TiO_2_/diatomite and BTD have strong absorption in the ultraviolet region. The absorption edge of TiO_2_ is about 390 nm. The absorption of BiOCl is mainly concentrated in the range of <380 nm, while the absorption edges of TiO_2_/diatomite and BTD show a small red shift compared with BiOCl and TiO_2_. The band gap energy (*E*_g_) of the samples was calculated by the Kubelka–Munk equation [44]: α*h*ν = *A*(*h*ν − *E*_g_)*^n^*^/2^, and the absorption coefficient, photon energy, a constant and band gap of the sample are expressed by α, *h*ν, *A* and *E*_g_, respectively. The calculated *E*_g_ of the samples is shown in Figure 8b. The *E*_g_ of pure BiOCl and TiO_2_ is about 3.09 eV and 3.18 eV, while the *E*_g_ of TiO_2_/diatomite and BTD is about 2.88 and 2.80 eV, respectively. The *E*_g_ of TiO_2_/diatomite is similar to previous reports [45] and significantly lower than ordinary TiO_2_ (3.18 eV). The *E*_g_ of BTD decreases, obviously due to the formation of heterojunction structures in the composite, which is beneficial to visible light response.

**Figure 9:**
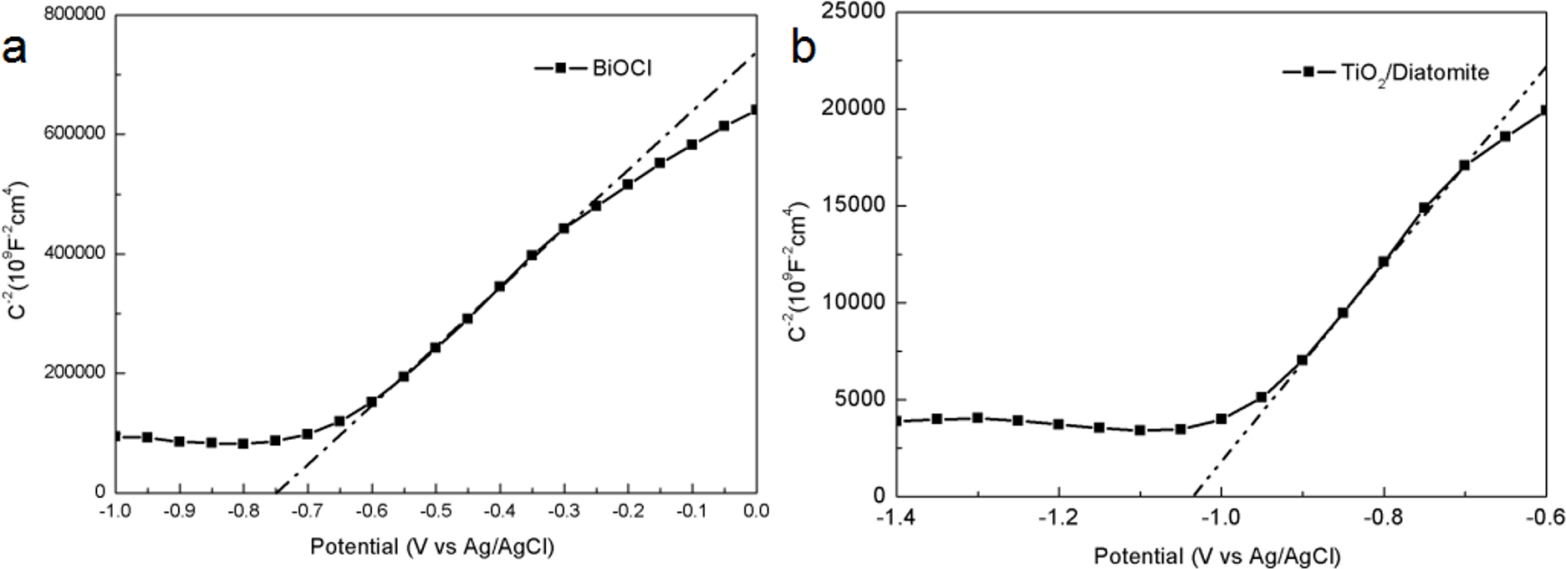
Mott–Schottky plots of BiOCl (a) and TiO_2_/diatomite (b).

To further study the band structure of the composites, the Mott–Schottky curves were calculated and are plotted in Figure 9. BiOCl and TiO_2_/diatomite are n-type semiconductors, and the flat-band potential (vs Ag/AgCl) is −0.75 V and −1.04 V, respectively. According to Equation 1, the flat-band potential relative to Ag/AgCl can be converted to the normal hydrogen electrode (NHE) potential:

[1] $$E_{\text{NHE}}=E_{\text{Ag/AgCl}}+E^{0}{}_{\text{Ag/AgCl}}\;,$$

where *E*^0^_Ag/AgCl_ = 0.197 V [46]. Generally, for n-type semiconductors, the flat-band potential is about 0.1 V smaller than the minimum of the conduction band (CB). Therefore, the positions of the CB for BiOCl and TiO_2_/diatomite are about −0.45 V and −0.74 V (vs NHE), respectively. According to Equation 2 and *E*_g_ of BiOCl and TiO_2_/diatomite:

[2] $$E_{\text{g}}=E_{\text{VB}}+E^{0}{}_{\text{CB}}\;.$$

Therefore, the positions of the valence band (VB) for BiOCl and TiO_2_/diatomite are 2.64 V and 2.14 V, respectively.

**Figure 10:**
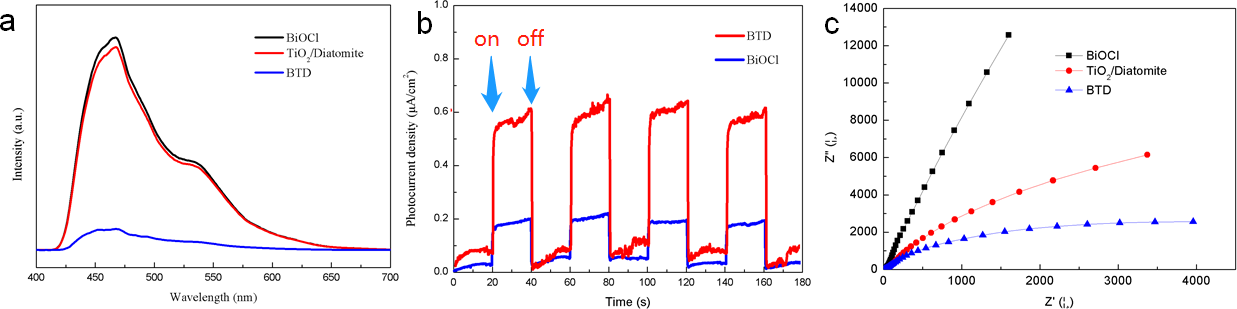
Photoluminescence spectra (a), transient photocurrent spectra (b) and electrochemical impedance spectra (c) of the samples.

The photocatalytic activity is determined not only by the band structure but also by the carrier transport efficiency [47]. In order to study the carrier migration efficiency of the samples, photoluminescence spectroscopy, photocurrent and electrochemical impedance spectroscopy were tested. The recombination rate of photogenerated carriers (electrons and holes) in photocatalysts was characterized by photoluminescence spectra. Generally, the lower the spectral intensity, the lower the recombination rate, and the better the photocatalytic performance. Although BTD only slightly reduces the band gap and enhances the light response ability, it has the lowest PL spectral intensity shown in Figure 10a, indicating that BTD can efficiently promote photogenerated electron–hole separation and transfer, which can significantly improve the photocatalytic activity.

Figure 10b shows the transient photocurrent response of the samples irradiated by visible light. The photocurrent is mainly generated by the migration and diffusion of photogenerated electrons and holes from the internal structure of the material to its surface [48]. BTD shows the most prominent photocurrent density, proving that the carrier recombination rate is low and the lifetime is long, which contributes to the enhancement of photocatalytic activity. Electrochemical impedance spectroscopy (EIS) is one of the most common methods to study the charge transfer efficiency of materials [49]. Generally, the smaller the radius of curvature in the EIS Nyquist diagram, the greater the charge transfer efficiency, which will effectively promote the photogenerated electron–hole separation. As shown in the EIS Nyquist diagram (Figure 10c), the impedance curve of the composite BTD has the smallest radius of curvature, in other words, it has the highest charge transfer efficiency and the highest utilization of photogenerated carriers. All of the above discussions show that BTD has a low electron–hole recombination rate and high carrier utilization rate with slightly reduced bandgap, which will effectively improve the photocatalytic activity.

**Figure 11:**
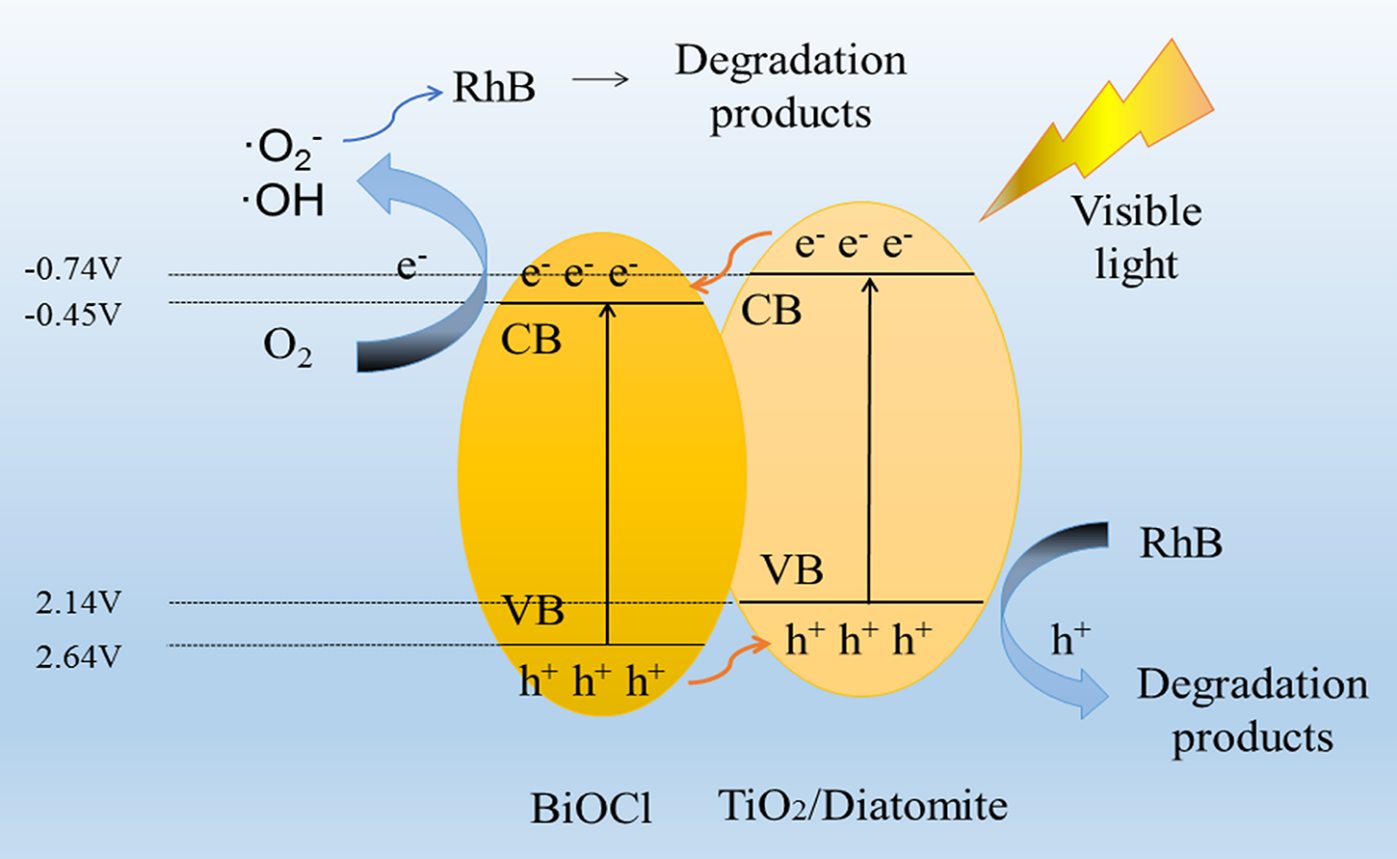
Photocatalytic mechanism of the BTD composite under visible-light irradiation to degrade RhB.

According to the discussion and analysis of the above results, the possible degradation mechanism of RhB by BTD composites was demonstrated in Figure 11. The result shows that BTD is a heterostructure. Under visible light, the composite is activated to generate electron–hole pairs [50]. The photoelectrons transfer from the CB of TiO_2_/diatomite to BiOCl, while the holes transfer from the VB of BiOCl to TiO_2_/diatomite. The transfer of carriers effectively inhibits the recombination of electron–hole pairs and improves photocatalytic performance. Next, the carriers move rapidly from inside the material to the surface. Subsequently, photogenerated electrons reduce O_2_ to generate ·O_2_^−^, and part of the ·O_2_^−^ will further generate OH with H_2_O. These active species, including ·O_2_^−^, OH and holes, have strong redox ability, and as a result, RhB can be degraded into small molecules to complete the photocatalytic process.

## Conclusion

In summary, a BiOCl/TiO_2_/diatomite (BTD) composite was synthesized by a modified sol–gel method and precipitation/calcination method. The prepared BTD composite exhibits excellent photocatalytic activity toward RhB degradation under visible-light irradiation. According to the characterization and analysis, the BTD composite has a heterojunction structure with an energy gap of 2.80 eV that can efficiently produce electrons and holes under illumination by visible light. Meanwhile, the heterojunction structure can promote the transfer of carriers, and can thus hinder the recombination of photogenerated electron–hole pairs. As a result, the photocatalytic activity is considerably improved. The BTD composite developed in this work has great application potential in the field of organic wastewater treatment driven by solar energy.

## Supporting Information

File 1 Additional figures.
