# Supplementary material for: BiOCl/TiO2/diatomite composites with enhanced visible-light photocatalytic activity for the degradation of rhodamine B
Source: Beilstein J Nanotechnol. 2019 Jul 16;10:1412–22. doi: 10.3762/bjnano.10.139 (PMC6664401; doi:10.3762/bjnano.10.139)
Supplement: File 1 — Additional figures. [file Beilstein_J_Nanotechnol-10-1412-s001.pdf]

BEILSTEIN JOURNAL OF NANOTECHNOLOGY

Supporting Information

for

$BiOCl/TiO_2$/diatomite composites with enhanced visible-light photocatalytic activity for the degradation of rhodamine B

Minlin Ao, Kun Liu, Xuekun Tang, Zishun Li, Qian Peng and Jing Huang

Beilstein J. Nanotechnol. **2019,** *10,* 1412–1422. doi:10.3762/bjnano.10.139

Additional figures

License and Terms: This is a supporting information file under the terms of the Creative Commons Attribution License (http://creativecommons.org/licenses/by/4.0). Please note that the reuse, redistribution and reproduction in particular requires that the authors and source are credited.
The license is subject to the *Beilstein Journal of Nanotechnology* terms and conditions: (https://www.beilstein-journals.org/bjnano)

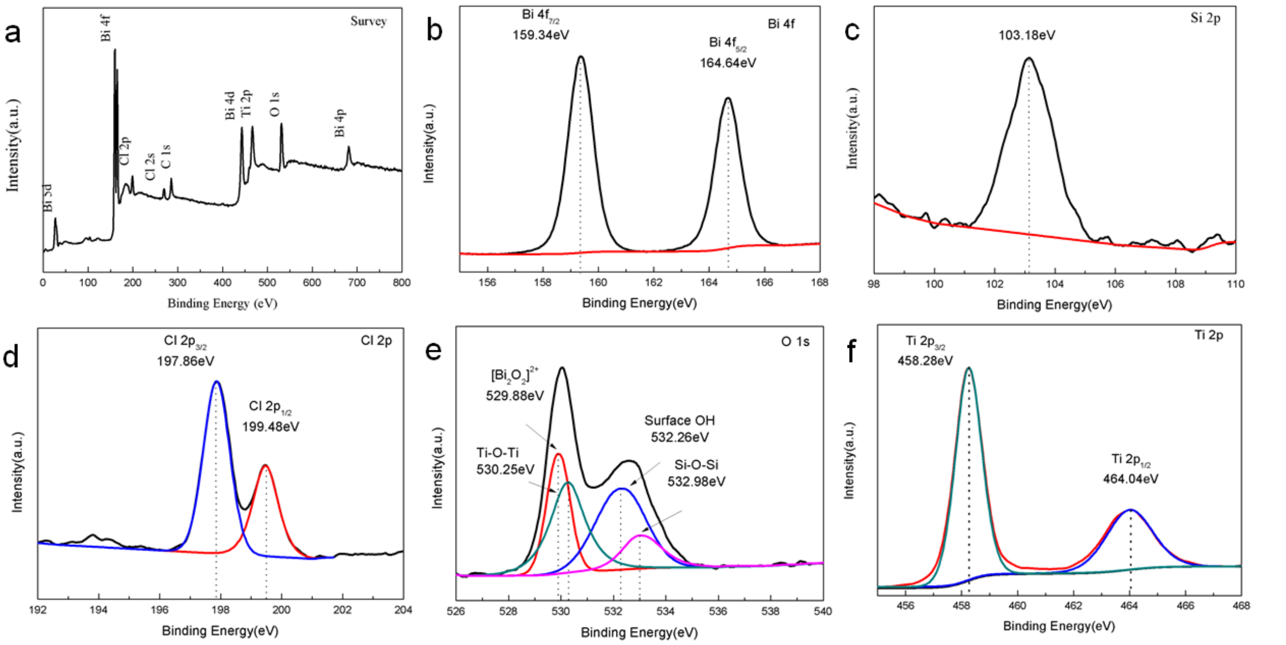

Figure S1: XPS spectra of recovered BTD, including survey scan (a), Bi 4f (b), Si 2p (c), Cl 2p (d), O 1s (e) and Ti 2p (f).

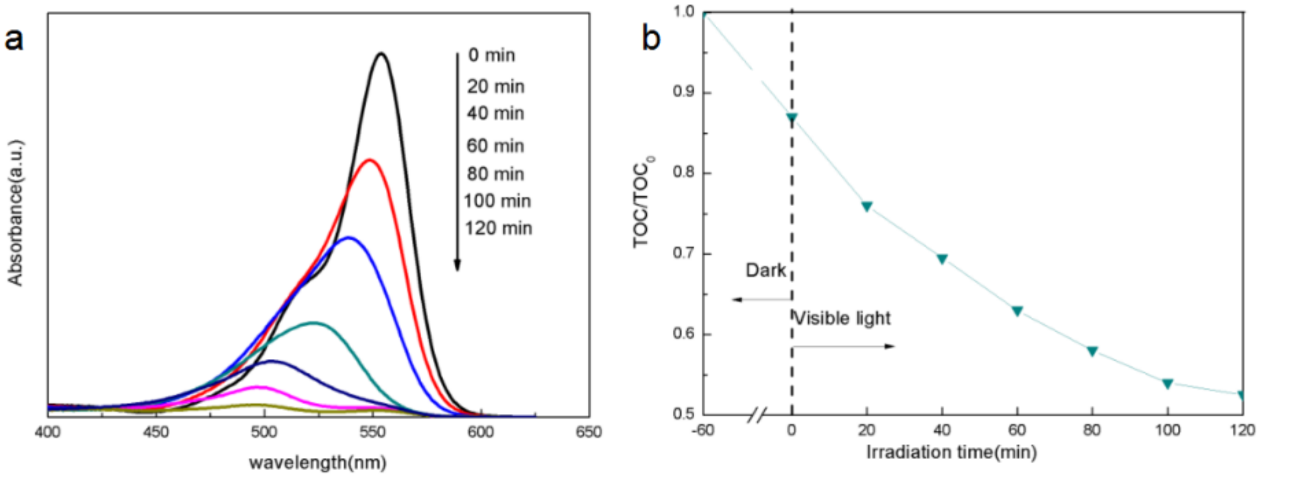

Figure S2: UV–vis spectra (a) and change chart of TOC (b) of RhB solution during photodegradation process.

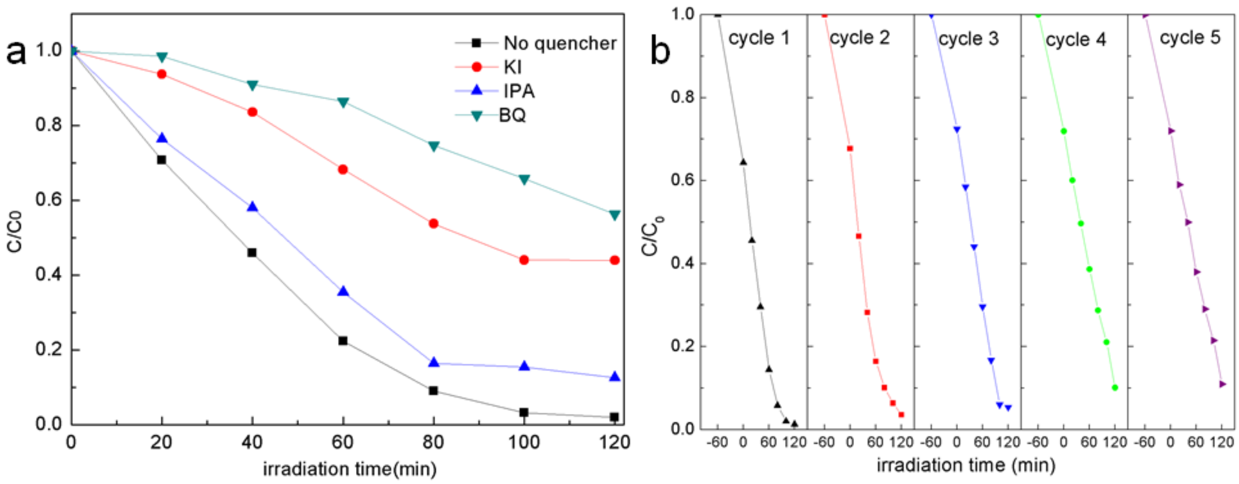

Figure S3: Free radical capture experiments (a) and cycling tests (b) for the photodegration of RhB with BTD.
